# Delayed-onset organizing pneumonia following perioperative COVID-19 after lobectomy

**DOI:** 10.1093/jscr/rjaf806

**Published:** 2025-10-07

**Authors:** Yasuhiro Nakashima, Shinji Katayanagi, Mariko Hanafusa, Hironori Ishibashi, Kei Aoyagi, Atsushi Nakagawa, Chika Noguchi, Hiroshi Hosoda

**Affiliations:** Department of Thoracic Surgery, Tokyo Kyosai Hospital, 2-3-8 Nakameguro, Meguro-ku, Tokyo 153-8934, Japan; Department of Thoracic Surgery, Institute of Science Tokyo, 1-5-45 Yushima, Bunkyo-ku, Tokyo 113-8519, Japan; Department of Respiratory Medicine, Tokyo Kyosai Hospital, 2-3-8 Nakameguro, Meguro-ku, Tokyo 153-8934, Japan; Department of Respiratory Medicine, Kamagaya General Hospital, 929-6 Hatsutomi, Kamagaya, Chiba 273-0121, Japan; Department of Thoracic Surgery, Institute of Science Tokyo, 1-5-45 Yushima, Bunkyo-ku, Tokyo 113-8519, Japan; Division of Cohort Research, National Cancer Center Institute for Cancer Control, 5-1-1 Tsukiji, Chuo-ku, Tokyo 104-0045, Japan; Department of Thoracic Surgery, Tokyo Kyosai Hospital, 2-3-8 Nakameguro, Meguro-ku, Tokyo 153-8934, Japan; Department of Thoracic Surgery, Institute of Science Tokyo, 1-5-45 Yushima, Bunkyo-ku, Tokyo 113-8519, Japan; Department of Respiratory Medicine, Tokyo Kyosai Hospital, 2-3-8 Nakameguro, Meguro-ku, Tokyo 153-8934, Japan; Department of Respiratory Medicine, Tokyo Kyosai Hospital, 2-3-8 Nakameguro, Meguro-ku, Tokyo 153-8934, Japan; Department of Respiratory Medicine, Tokyo Kyosai Hospital, 2-3-8 Nakameguro, Meguro-ku, Tokyo 153-8934, Japan; Department of Thoracic Surgery, Tokyo Kyosai Hospital, 2-3-8 Nakameguro, Meguro-ku, Tokyo 153-8934, Japan

**Keywords:** COVID-19, organizing pneumonia, post-acute COVID-19 syndrome, postoperative complications, pleurodesis, thoracic surgery

## Abstract

Delayed-onset organizing pneumonia as a manifestation of post-acute coronavirus disease 2019 (COVID-19) syndrome has not been documented in the perioperative setting. Here, a 61-year-old man underwent left lower lobectomy complicated by persistent air leakage requiring seven pleurodesis procedures. He developed COVID-19 on postoperative Day 10 and initially recovered but was readmitted on Day 27 with fever and respiratory failure. Chest computed tomography revealed progressive consolidations with ground-glass opacities. Initial methylprednisolone pulse therapy showed limited response, necessitating a second course with cyclosporine A addition. The patient achieved substantial radiological improvement by postoperative Day 104. Based on the biphasic clinical course, distinctive radiological progression, and limited steroid response, delayed-onset organizing pneumonia secondary to post-acute COVID-19 syndrome was diagnosed. This case highlights the importance of extended monitoring in post-lung resection patients with COVID-19 to enable early recognition and prompt intervention of delayed pulmonary complications.

## Introduction

Perioperative coronavirus disease 2019 (COVID-19) infection significantly increases postoperative morbidity and mortality, particularly severe pulmonary complications [1]. Although acute complications of severe perioperative COVID-19 are well-documented, post-acute COVID-19 syndrome following mild perioperative COVID-19 infections remains incompletely characterized. Among emerging post-COVID-19 interstitial lung complications, delayed-onset organizing pneumonia (OP) represents a distinct clinical entity where respiratory symptoms initially improve before deteriorating weeks after initial clinical recovery [2–4]. Herein, we report the first documented case of delayed-onset OP following recovery from mild COVID-19 pneumonia during the perioperative period of pulmonary surgery. This case highlights the importance of extended monitoring in post-lung resection patients with COVID-19 to enable early recognition and prompt intervention of delayed pulmonary complications.

## Case report

**Figure 1:**
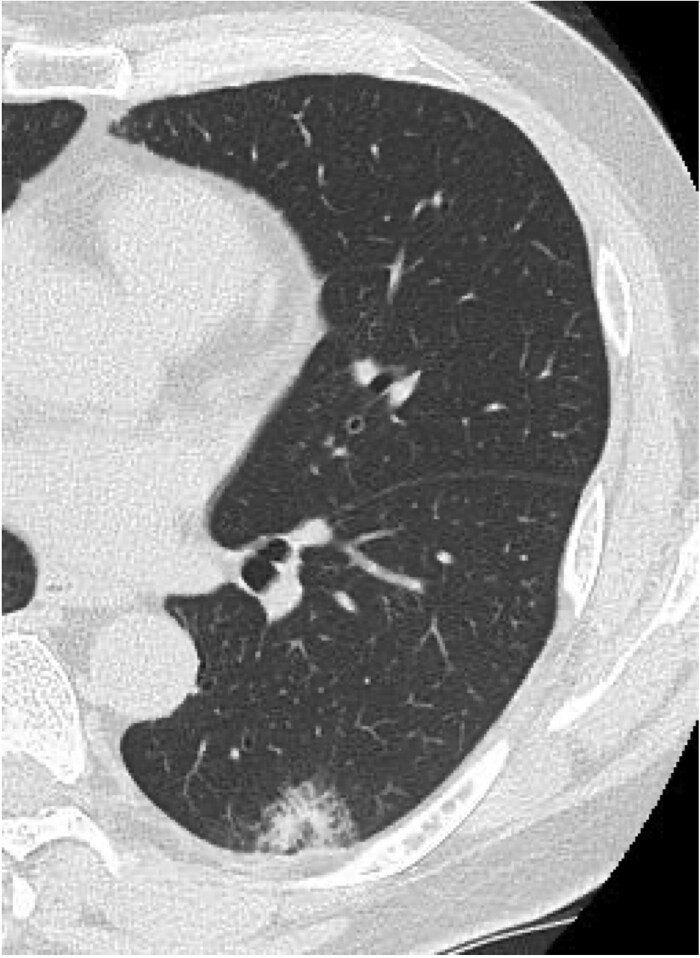
Preoperative chest computed tomography showing a 35 × 12 × 20 mm nodule with mild ground-glass opacity in the left lower lobe (S10).

A 61-year-old man underwent left lower lobectomy for a 3.5-cm peripheral adenocarcinoma via single-port video-assisted thoracic surgery (Fig. 1). He was a former smoker (20 pack-years, quit 21 years ago) and had received his second COVID-19 vaccination 4 months prior. His postoperative course was complicated by persistent air leakage, requiring 7 pleurodesis procedures, including minocycline (5 attempts), autologous blood (once), and OK-432 with minocycline (once). The air leak resolved on postoperative day (POD) 9, and the chest tube was removed on POD 10.

**Figure 2:**
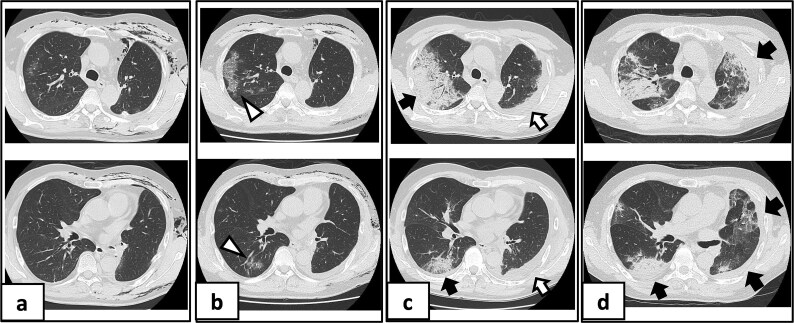
Serial chest imaging demonstrating pulmonary complications. (a) Postoperative day (POD) 10: Initial presentation without significant abnormalities. (b) POD 16: Peripheral ground-glass opacities (arrowheads). (c) POD 27: Patchy consolidation (solid arrows) with new ground-glass opacities and pleural effusion (open arrows). (d) POD 36: Progressive consolidation (solid arrows).

On POD 10, he developed fever and tested positive for COVID-19 by reverse transcription-polymerase chain reaction. Nosocomial transmission was suspected given recent ward cases during the delta variant outbreak. Chest computed tomography showed minimal peripheral ground-glass opacities in the right upper lobe (Fig. 2a and b). He received remdesivir (5 days) and dexamethasone (7 days), maintained adequate oxygenation without supplemental oxygen, and was discharged on POD 21 after clinical improvement.

On POD 27, he was readmitted with fever (38.2°C) and respiratory failure. COVID-19 testing was negative. Chest computed tomography revealed patchy consolidations mixed with ground-glass opacities in the right upper lobe with notable expansion, and new ground-glass opacities in the right middle lobe and residual left upper lobe periphery (Fig. 2c). Findings excluded recurrent air leak or aspiration pneumonia. Despite antibiotic therapy, oxygen requirements increased to 2 L by POD 29.

**Figure 3:**
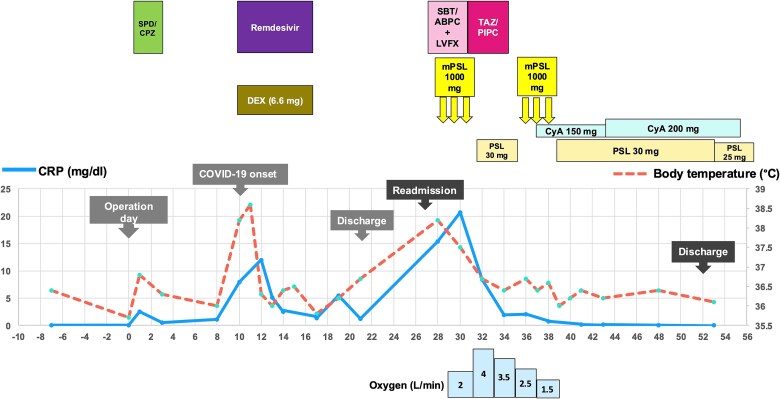
Clinical course depicting body temperature (°C, dashed line) and serum CRP levels (mg/dl, solid line). Upper panel outlines treatment regimens including antibiotics (SPD/CPZ: Sulbactam/cefoperazone; SBT/ABPC: Sulbactam/ampicillin; LVFX: Levofloxacin; TAZ/PIPC: Tazobactam/piperacillin), corticosteroids (DEX: Dexamethasone; mPSL: Methylprednisolone; PSL: Prednisolone), and immunosuppressants (CyA: Cyclosporine A). The lower panel shows oxygen requirements (L/min) via nasal cannula.

**Figure 4:**
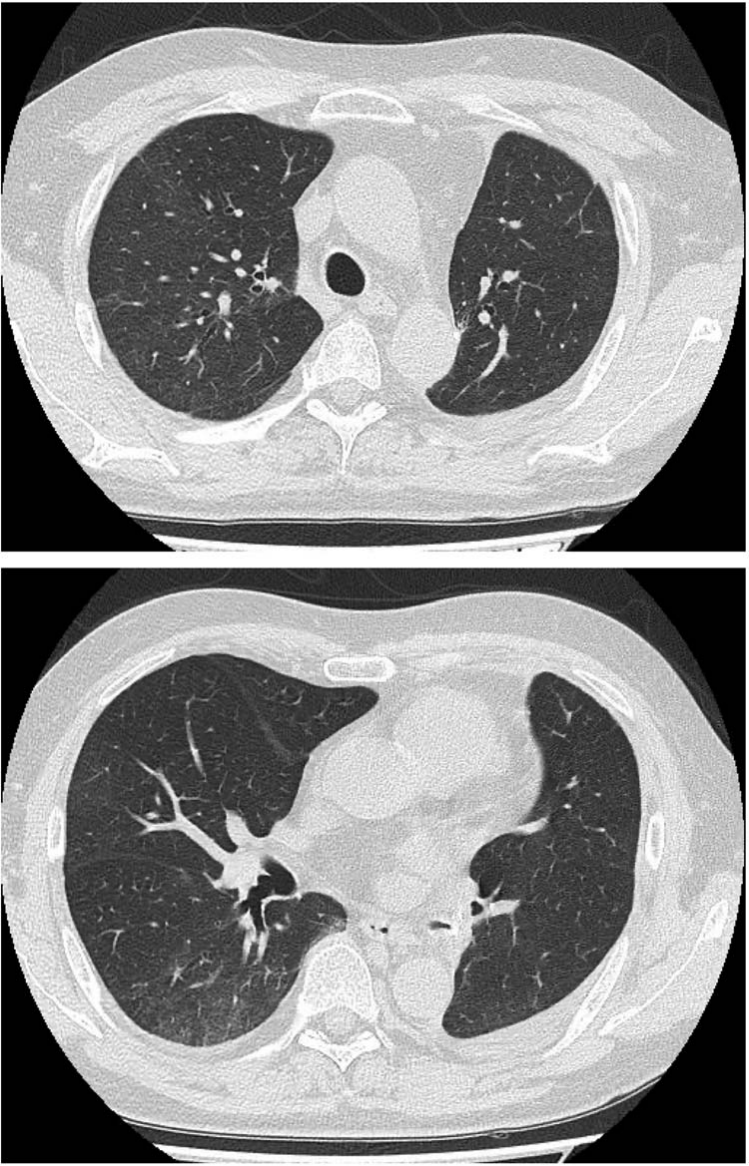
Follow-up chest imaging on postoperative Day 104 showing significant resolution of bilateral consolidations observed during the acute phase of organizing pneumonia.

Methylprednisolone pulse therapy (1 g/day for 3 days) was initially administered, followed by oral prednisolone (30 mg). However, oxygen requirements increased to 4 L on POD 31 (Fig. 3). On POD 36, a second methylprednisolone pulse course was initiated with cyclosporine A (150 mg) owing to progressive consolidation on chest imaging (Fig. 2d). Oxygen support was discontinued by POD 38, and he was discharged on POD 52. Follow-up image on POD 104 showed substantial radiological improvement (Fig. 4). Immunosuppressive medications were successfully tapered and discontinued by POD 329. This case was diagnosed as delayed-onset OP as a manifestation of post-acute COVID-19 syndrome, characterized by the biphasic clinical course and distinctive radiological progression occurring weeks after acute COVID-19 infection.

## Discussion

Previous reports of perioperative COVID-19 infection following pulmonary surgical procedures have focused primarily on severe acute phase outcomes [5]. Delayed-onset OP as a manifestation of post-acute COVID-19 syndrome has not been documented, highlighting an important knowledge gap in postoperative complications.

Recognizing secondary OP remains challenging for surgeons when patients who recovered from mild perioperative COVID-19 present with clinical deterioration upon readmission. Although minocycline-induced acute eosinophilic pneumonia (AEP) was initially considered in our case due to minocycline exposure during pleurodesis, several clinical features argued against this diagnosis: the biphasic course, absence of peripheral eosinophilia, and limited initial steroid response. Minocycline-induced AEP typically follows an acute monophasic pattern, develops peripheral eosinophilia during hospitalization, and demonstrates rapid clinical improvement within 24–48 hours of steroid initiation [6].

Typical COVID-19-related lung changes peak between Days 9 and 13 [7]. However, secondary OP following COVID-19 in non-surgical patients demonstrates a distinct biphasic pattern, with initial improvement after short-term steroid therapy followed by a second respiratory deterioration peak between Days 28 and 60 after the COVID-19 diagnosis [2–4]. The mixed ground-glass opacities and consolidations during relapse are characteristic imaging findings [3, 4]. Unlike organizing pneumonia in severe COVID-19 with diffuse alveolar damage, this entity achieves complete radiological resolution over months rather than irreversible fibrosis [3, 4]. Our case demonstrated a biphasic clinical course consistent with these reports. The patient showed good initial treatment response but developed clinical deterioration on POD 27 (17 days after COVID-19 diagnosis), with consolidations expanding beyond 4 weeks and substantial improvement achieved at 3 months post-onset.

Secondary OP requires high doses of corticosteroid therapy over a prolonged treatment duration, typically weeks to months, as 10 days of dexamethasone might not be sufficient treatment [8]. Relapse is not uncommon, affecting 13% to 68% of cases, with delayed treatment initiation at the time of initial OP diagnosis increasing this risk [8].

This case was complicated by pre-existing thoracic inflammation from both surgical trauma and multiple pleurodesis procedures prior to COVID-19 infection. Although OK-432 pleurodesis has been associated with interstitial lung disease development [9], the role of prior pleurodesis in predisposing to COVID-19-related organizing pneumonia remains speculative. The earlier onset at 17 days after initial COVID-19 diagnosis, compared to the typical 28–60-day interval reported in non-surgical patients [2–4], and the need for additional immunosuppression with cyclosporine A may reflect this complex inflammatory background. However, the exact relationship between perioperative inflammation and subsequent secondary OP remains poorly characterized due to limited available data.

This case highlights the importance of extended monitoring for delayed pulmonary complications in post-lung resection patients with COVID-19. When patients present with biphasic deterioration patterns, early recognition of secondary OP is important. Our experience suggests that conventional short-term steroid regimens are insufficient and prolonged immunosuppressive therapy may be required.

## Data Availability

The datasets supporting the conclusions of this article are included in Figs 1–4.

## Conference presentation

The subject matter of this case study was presented at the 40^th^ Annual Meeting of the Japanese Association for Chest Surgery, Niigata, Japan, on July 13–14, 2023.
